# Clinical Profiles, Characteristics, and Outcomes of the First 100 Admitted COVID-19 Patients in Pakistan: A Single-Center Retrospective Study in a Tertiary Care Hospital of Karachi

**DOI:** 10.7759/cureus.8712

**Published:** 2020-06-20

**Authors:** Muhammad Sohaib Asghar, Syed Jawad Haider Kazmi, Noman Ahmed Khan, Mohammed Akram, Salman Ahmed Khan, Uzma Rasheed, Maira Hassan, Gul Muhammad Memon

**Affiliations:** 1 Internal Medicine, Dow International Medical College, Dow University Hospital, Dow University of Health Sciences, Karachi, PAK; 2 Emergency Medicine, Liaquat National Hospital and Medical College, Karachi, PAK; 3 General Surgery, Liaquat National Hospital and Medical College, Karachi, PAK; 4 Internal Medicine, Liaquat National Hospital and Medical College, Karachi, PAK; 5 Internal Medicine, Dow International Medical College, Karachi, PAK

**Keywords:** coronavirus, covid-19, intensive care unit, mortality, severity, markers, indices, indicators, pandemic, pakistan

## Abstract

Background and objective

COVID-19 is a highly disseminating viral disease imparted by severe acute respiratory syndrome coronavirus 2 (SARS-COV-2), which was declared a global pandemic by the World Health Organization. In our study, we aimed to describe the clinical characteristics of the first 100 hospitalized patients of confirmed COVID-19 in a developing country.

Materials and methods

The study included all the admitted patients (n = 100) having COVID-19 polymerase chain reaction (PCR) positive, and evaluated clinical profiles and characteristics of the patients linking to disease severity.

Results

Out of the 100 patients, 67 were in the ward, 33 were in ICU, 78 of them recovered, while 22 deaths reported. The mean age was 52.58 ± 15.68, with most frequent comorbidities are diabetes and hypertension while frequent symptoms are fever and dry cough. Bilateral lower zone patchy infiltrates are frequent chest radiographic findings. Amongst the patients admitted in ICU, there were significant differences in the total leukocyte count (P = 0.001), neutrophils and lymphocytes (P =< 0.001), monocytes (P = 0.027), urea (P =< 0.001), creatinine (P = 0.002), neutrophil-to-lymphocyte ratio (NLR) and platelet-to-lymphocyte ratio (PLR) increasing with disease severity, lymphocyte-to-monocyte ratio (LMR) and lymphocyte-to-C‐reactive protein ratio (LCR) decreasing with mortalities. Gamma-glutamyl transferase (GGT) followed by aspartate aminotransferase (AST) are frequent hepatic derangements, while C-reactive protein (CRP) levels predicting ICU admission with area under the curve (AUC): 0.806, positive predictive value (PPV): 85.1% and lactate dehydrogenase (LDH) predicting mortality with AUC: 0.877, PPV: 97.3%, while NLR (AUC: 0.806, PPV: 95.8%) for mortality and neutrophils (AUC: 0.773, PPV: 87.5%) for ICU patients.

Conclusion

A number of factors are linked with disease severity and mortality along with dynamic changes of the laboratory investigations during hospital stay affecting prognosis.

## Introduction

Corona is a highly disseminating viral disease imparted by severe acute respiratory syndrome coronavirus 2 (SARS-COV-2); virus depicts strong phylogenetic relativity to severe acute respiratory syndrome bat virus (SARS- bat virus). Round or elliptical shaped SARS-COV-19 is a positive-stranded RNA virus belonging to betaCoV genus of family Coronaviridae, with bats and rodents as primary reservoirs [1-2]. Six species of family Coronaviridae are identified to infect the human race comprising two zoonotic viruses: 1) Severe acute respiratory syndrome coronavirus (SARS-COV), and 2) Middle-East respiratory syndrome (MERS-COV), accountable for major epidemics of China in 2002-2003, and Middle East in 2012 [3]. SARS-COV-2 depicts pathogenicity of infiltrating epithelial cells of the human respiratory tract in lieu of communication between viral S protein and angiotensin-converting enzyme 2 (ACE 2) on the surface of epithelial cells thus retaining a high efficiency in infecting human race [4]. A wide range of zoonotic animals comprising camels, cats, cattle, and bats are liable to disseminate coronaviruses; in regards with human to human transmission, intimate contact with the sufferer is responsible for inhaling the contaminated respiratory droplets by cough and sneezing. This dissemination is encountered in both hospital and family setups [5-6]. Coronavirus can survive up to nine days on inanimate surfaces spreading infection by inoculating itself in cells of nose, ears, and mouth [6]. In the concluding days of 2019, Wuhan, a business originating city of China, had an epidemic of novel coronavirus with mortality of more than 1800 and infected 70,000 inhabitants in the initial 50 days [1]. World Health Organization (WHO) has since stated coronavirus (COVID-19) as a mass emergency of international burden with 7,544,790 laboratories proved cases registered worldwide (assessed on 11th June 2020). The global rate of deaths documented is 421,556 while patients recovered from manifestations of coronavirus documented are 3,823,429 [7].

Every age group is prone to suffer from the novel coronavirus. But according to data received from studies conducted throughout the world, frequently affected individuals belong to middle and older age groups with an age range of 65-85 years [8]. Age group infected by a novel coronavirus in China was 10 to ≥80 years with a median age of 30-79 years suffering frequently [9-10]. Italy reported an age group of 18 to ≥70 years with a median age of ≥50 years, while the USA being the most affected country globally, reported age range of 19 to ≥85 with a median age of 65-84 years. Coronavirus has slightly increased gender affinity towards males as compared to females globally [4-5, 11]. Novel coronavirus disease (COVID-19) has wide parameters of clinical manifestations ranging from asymptomatic carriers of the disease to manifestations with respiratory failure requiring mechanical ventilation to ICU (intensive care unit) supports, multi-organ and systemic symptoms ranging from sepsis, septic shock and multiple organ dysfunction syndromes (MODS) [2]. Based on severity, manifestations of coronavirus are mild category encountered by majority with no or mild pneumonia manifestations, moderate and severe with sepsis, severe pneumonia, and MODS [2]. Most frequently encountered manifestations in initial onset of infection were fever (98.6%), fatigue (69.6%), dry cough (59.4%), muscle pain (34.8%), dyspnea (31.2%), and least frequently suffered symptoms were headache (6.5%), dizziness (9.4%), abdominal pain (2.2%), diarrhea (10.1%), nausea (10.1%) and vomiting (3.6%) [7, 11]. Coronavirus as quoted by many studies usually infects the older population, the most characteristic comorbidities encountering infection are hypertension (15%), diabetes (12%), cardiovascular disorders (10%), and cerebrovascular disorders (7%) [3-4, 12-13]. The WHO endorses acquiring samples from both the upper respiratory tract (nasal and oropharyngeal swabs) and lower respiratory tract (expectorated sputum, endotracheal aspirate, and bronchoalveolar lavage). Samples ought to be preserved at 4 degrees celsius. These samples of saliva and mucus are analyzed by reverse transcription polymerase chain reaction (RT-PCR) amplifying genetic material obtained from samples and detection of CoV material is done and preserved [2].

Laboratory findings detected in the majority of COVID-19 disease sufferers are lymphocytopenia (82.3%), thrombocytopenia (36.2%), leukopenia (33.7%), majority of patients reported high levels of C-reactive protein (CRP), less frequent were high levels of alanine transaminase (ALT), aspartate aminotransferase (AST), creatinine kinase (CK) and D-dimer [2, 14]. Computed tomography scans (CT-Scan) in a majority of patients is suggestive of ground-glass opacity (65%), ill-defined margins (81%), smooth or irregular interlobular septal thickening (35%), air bronchogram (47%), crazy paving pattern (10%) and thickening of neighboring pleura (32%) [4]. There are no definitive antivirals and vaccinations introduced in recent intervals to treat novel coronavirus disease (COVID-19), so a prominent regimen in the current scenario is supportive care comprising of broad-spectrum antibiotics, antivirals, corticosteroids, oxygen therapy, mechanical ventilation and convalescent plasma [3, 5, 14-15].

The study aimed to describe all clinical and biochemical characteristics of patients suffering from COVID-19 as well as identifying the markers that are suggestive of poor prognosis and mortality in patients of COVID-19. The study focused on all the hospital admissions, correlating laboratory investigations with the severity of the disease, and to our knowledge, it is the first study of such kind in this region.

## Materials and methods

This study was conducted as a single centered, retrospective, observational study, during the months of March and April 2020, and including all patients who were diagnosed as COVID-19 positive via either nasopharyngeal or oropharyngeal swab for PCR. The diagnostic kit used exploits the principle of real-time fluorescence (RT-PCR), USA-WA1/2020 stock concentration 2.8E+05 TCID50/mL, with a lower detection limit of 0.003 TCID50/mL. The sensitivities of RT-PCR via using nasal and oropharyngeal swabs have previously been discussed by Wang et al. [16]. Nasal swabs were used in 44% while oral swabs diagnosed 56% of the study population. Once admitted, the patients were monitored for their disease course and the outcome was followed subsequently. Two-thirds of the patients were admitted to the ward with mild signs and symptoms, while the rest one-third showed moderate to severe disease progress in ICU. Out of the total 100 patients included, 57 were recovered and discharged with two consecutive negative PCR for Covid-19, 21 patients who remained asymptomatic were discharged for home isolation early without PCR being negative. A total of 22 deaths reported due to COVID-19, out of whom 19 patients were admitted to ICU for critical care management and ventilators.

The statistical analysis was conducted by the Statistical Package for the Social Sciences (SPSS version 25.0, IBM Corp., Armonk, NY). All continuous variables were described as both mean and standard deviation as well as median and interquartile range. The means were then compared using both independent sample t-test and Mann-Whitney U-test. The comparison of categorical data was done either using the Chi-square test or Fisher’s exact test. A p-value of <0.05 was considered statistically significant (2-tailed). In a paired sample t-test, each subject or entity was measured twice, resulting in pairs of observations for the progress of laboratory investigations at admission and discharge. A receiver operating characteristic (ROC curve) was also obtained to determine the predictive parameters for the severity of the disease.

## Results

One-half of the study population belonged to age group 50-75 years, with a mean of 52.58 years, which was significantly higher in severe patients (p < 0.001), but slightly lower in females (p = 0.211). Two-thirds of the study population comprised of males, with no disparity amongst gender in ICU admissions (34.8% vs 29.0%, p = 0.572), and deaths (23.2% vs 19.4%, p = 0.613). The rest of the demographic data is stated in Table 1. The frequent co-morbidities reported in the medical records of the patients were diabetes, hypertension, ischemic heart disease followed by chronic kidney disease, and chronic liver disease. The most frequent symptoms were fever (83%) and dry cough (52%) as shown in Table 2. Gastrointestinal manifestations were only exhibited by 17% of the patients. One-half of the patients presented with low-grade fever, and dyspnea was a feature of 43% subjects, around 25% required no need for respiratory support, 10% were invasively supported, 17% needed non-invasive bilevel positive airway pressure (BiPAP) support, while a majority of the patients were managed with supplemental oxygen and nebulization.

**Table 1:** Demographic data of the study population. * Indicates independent sample t-test; ** Indicates Chi-square test; ^ Indicates Mann-Whitney U-test; ” Indicates Fisher’s exact test. M: males; F: females; IHD: Ischemic heart disease; CVA: Cerebrovascular accident (Stroke); CKD: Chronic kidney disease; CLD: Chronic liver disease; COPD: Chronic obstructive pulmonary disease; T.B.: Tuberculosis.

Demographic data of the study population (n = 100)						p-value
1	Age (in years)	Age group	Frequency		Percent	0.423**
		<25	2		2.0	
		25-50	40		40.0	
		50-75	53		53.0	
		>75	5		5.0	
		Total	100		100.0	
2	Mean age (in years)	52.58 ± 15.68				-
		Males: 53.89 ± 15.30		Females: 49.64 ± 16.37		0.211*
		Ward: 48.49 ± 16.58		ICU: 60.87 ± 9.37		<0.001*
		Recovered: 50.14 ± 16.15		Death: 61.22 ± 10.08		<0.001*
3	Median age (IQR)	54.00 (40.25 – 65.00)				-
		Males: 55.00 (43.00 – 66.50)		Females: 54.00 (38.00 – 64.00)		0.291^^^
		Ward: 48.00 (36.00 – 60.00)		ICU: 63.00 (55.00 – 67.50)		<0.001^^^
		Recovered: 50.50 (38.00 – 63.25)		Death: 61.50 (56.00 – 67.75)		0.003^^^
3	Gender	Males: 69.0% (n = 69)		Females: 31.0% (n = 31)		0.572**
4	Hospital Stay	Ward: 67.0% (n = 67)		ICU: 33.0% (n = 33)		
5	Comorbidities	Diabetes: 41.00% (n = 41)		M: 43.47% (n = 30)	F: 35.48% (n = 11)	0.452**
		Hypertension: 32.00% (n = 32)		M: 31.88% (n = 22)	F: 32.25% (n = 10)	0.970**
		IHD: 13.00% (n = 13)		M: 15.94% (n = 11)	F: 6.45% (n = 2)	0.334”
		CVA: 3.00% (n = 3)		M: 2.89% (n = 2)	F: 3.22% (n = 1)	1.000”
		CKD: 10.00% (n = 10)		M: 7.24% (n = 5)	F: 16.12% (n = 5)	0.277”
		CLD: 9.00% (n = 9)		M: 5.79% (n = 4)	F: 16.12% (n = 5)	0.131”
		COPD: 3.00% (n = 3)		M: 2.89% (n = 2)	F: 3.22% (n = 1)	1.000”
		Asthma: 2.00% (n = 2)		M: 1.44% (n = 1)	F: 3.22% (n = 1)	0.526”
		Hypothyroidism: 4.0% (n = 4)		M: 1.44% (n = 1)	F: 9.67% (n = 3)	0.087”
		T.B: 1.00% (n = 1)		M: 1.44% (n = 1)	F: 0.0% (n = 0)	1.000”
7	Travel History	Present = 12 (12.00%)		Absent = 88 (88.00%)		-
	Occupation	Medical = 9 (9.00%)		Non-medical = 91 (91.00%)		
	Diagnosis	Nasopharyngeal PCR: 44.00%		Oropharyngeal PCR: 56.00%		

**Table 2:** Clinical profiles, symptomatology, and radiology of the study population (n = 100). CXR: Chest X-ray; ARDS: Acute respiratory distress syndrome.

Characteristics	Variables	Present	Absent
Symptomatology (n = 100)	Fever	n = 83 (83.0%)	n = 17 (17.0%)
	Dry cough	n = 52 (52.0%)	n = 48 (48.0%)
	Cough with sputum	n = 18 (18.0%)	n = 82 (82.0%)
	Sore throat	n = 16 (16.0%)	n = 84 (84.0%)
	Pleurisy	n = 10 (10.0%)	n = 90 (90.0%)
	Dyspnea	n = 43 (43.0%)	n = 57 (57.0%)
	Lethargy	n = 11 (11.0%)	n = 89 (89.0%)
	Fatigue	n = 16 (16.0%)	n = 84 (84.0%)
	Rhinitis	n = 15 (15.0%)	n = 85 (85.0%)
	Headache	n = 8 (8.0%)	n = 92 (92.0%)
	Decreased appetite	n = 5 (5.0%)	n = 95 (95.0%)
	Arthralgia/Myalgia	n = 14 (14.0%)	n = 86 (86.0%)
	Vomiting	n = 7 (7.0%)	n = 93 (93.0%)
	Nausea	n = 6 (6.0%)	n = 94 (94.0%)
	Diarrhea	n = 17 (17.0%)	n = 83 (83.0%)
	Abdominal pain	n = 5 (5.0%)	n = 95 (95.0%)
Grading of fever (n = 83)	99-100°F	n = 52 (62.65%)	n = 31 (37.34%)
	101-103°F	n = 28 (33.73%)	n = 55 (66.26%)
	>104°F	n = 3 (3.61%)	n = 80 (96.38%)
Respirator	Vent	n = 10 (10.0%)	n = 90 (90.0%)
	Bipap (NIV)	n = 17 (17.0%)	n = 83 (83.0%)
	Oxygen by mask	n = 30 (30.0%)	n = 70 (70.0%)
	High flow nasal oxygen	n = 6 (6.0%)	n = 94 (94.0%)
	Nebulization	n = 12 (12.0%)	n = 88 (88.0%)
	No need	n = 25 (25.0%)	n = 75 (75.0%)
CXR findings	Normal	n = 21 (21.00%)	n = 79 (79.00%)
	Consolidation	n = 16 (16.00%)	n = 84 (84.00%)
	Ground glass appearance	n = 19 (19.00%)	n = 81 (81.00%)
	Nodular opacity	n = 6 (6.0%)	n = 94 (94.0%)
	Pleural effusion	n = 12 (12.0%)	n = 88 (88.0%)
	ARDS	n = 7 (7.0%)	n = 93 (93.0%)
	Interstitial shadowing	n = 27 (27.0%)	n = 73 (73.0%)
	Patchy infiltrates	n = 39 (39.0%)	n = 61 (61.0%)
Zonal predominance	Upper zone	n = 5 (5.0%)	n = 95 (95.0%)
	Middle zone	n = 17 (17.0%)	n = 83 (83.0%)
	Lower zone	n = 57 (57.0%)	n = 43 (43.0%)
Location of patch	Central	n = 39 (39.0%)	n = 61 (61.0%)
	Peripheral	n = 28 (28.0%)	n = 72 (72.0%)
	Both	n = 22 (22.0%)	n = 78 (78.0%)
Laterality	Unilateral	n = 25 (25.0%)	n = 75 (75.0%)
	Bilateral	n = 54 (54.0%)	n = 46 (46.0%)
Mean no. of comorbidities per person: 0.98 ± 1.19		Mean symptoms per individual: 2.92 ± 1.41	
Mean days to develop dyspnea after onset: 4.52 ± 2.60		Mean days of hospital stay: 8.41 ± 6.00	

The mean days to develop dyspnea after the onset of symptoms was 4.52 ± 2.60 days, with a slightly decreased onset in deceased patients 3.40 ± 1.81 days (p = 0.358). The mean days of hospital stay was significantly more in ICU stay 12.24 ± 6.89 versus 6.52 ± 4.48 days in the ward (p < 0.001), while recovered patients (10.50 ± 5.78 days) stayed slightly more in hospital than deceased patients (8.90 ± 4.91 days) which was statistically insignificant (p = 0.257). The chest radiographs were used as the only imaging modality for the involvement of lungs showing predominantly bilateral lower zone patchy infiltrates or interstitial shadowing in more than half of the patients, while ground-glass (19%), consolidation (16%), effusion (12%), and acute respiratory distress syndrome (ARDS) (7%) were also reported. The results of laboratory investigations of the study population are as follows:

1) Hemogram (Complete Blood Picture)

**Figure 1:**
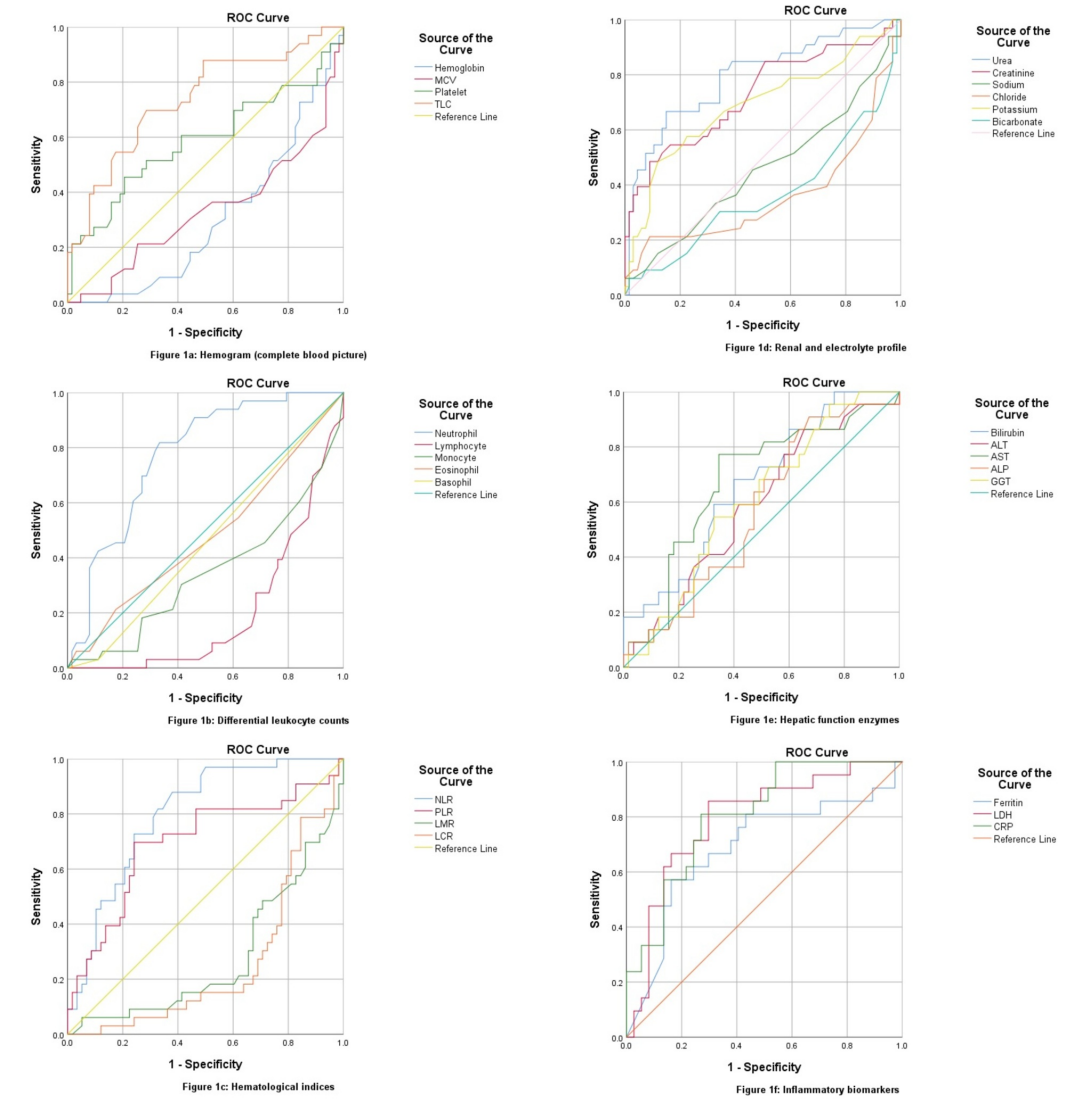
Receiver operating characteristic (ROC) curves of admitting laboratory investigations for predicting ICU stay.

**Figure 2:**
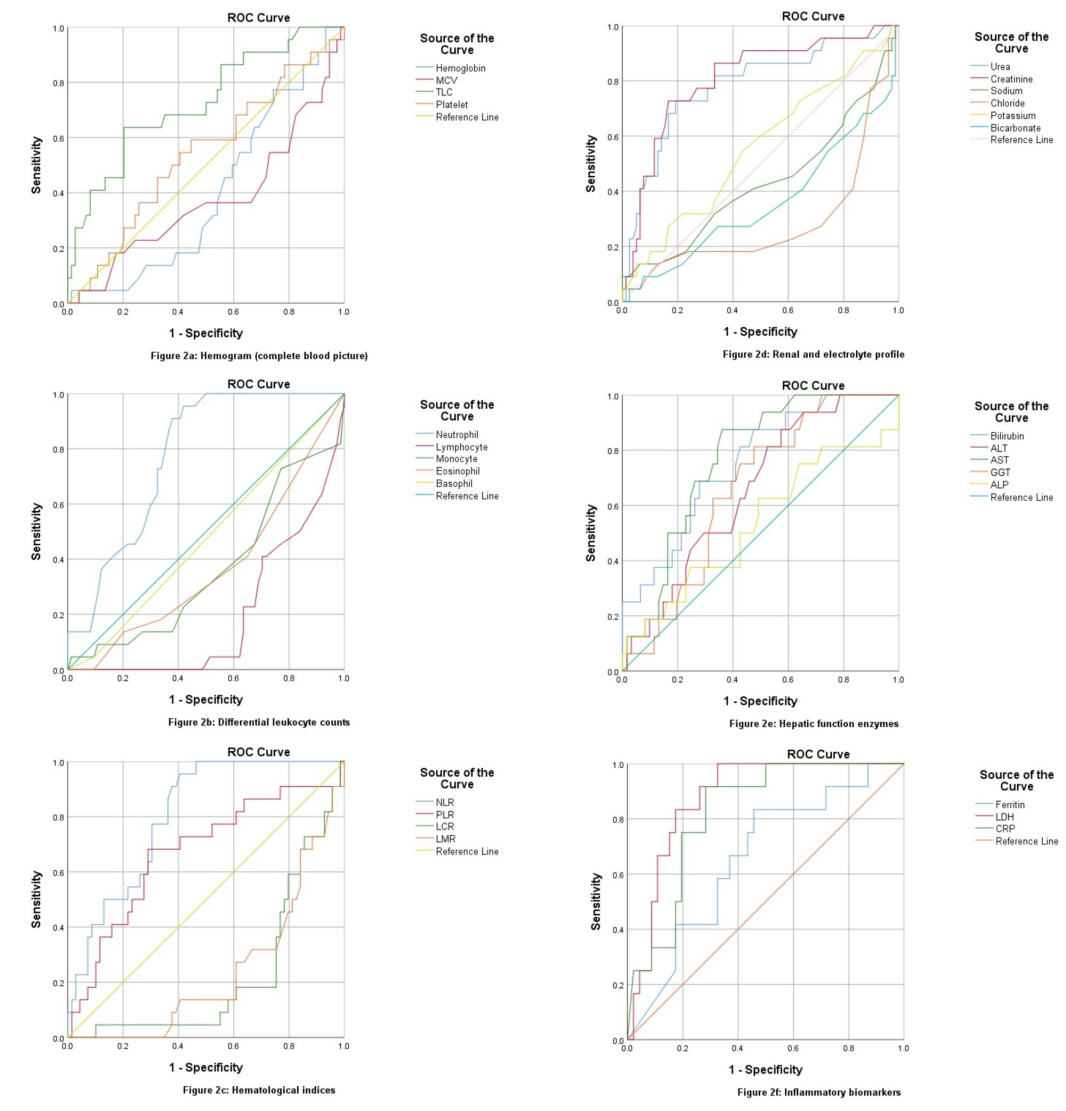
Receiver operating characteristic (ROC) curves of admitting laboratory investigations for predicting mortality.

**Figure 3:**
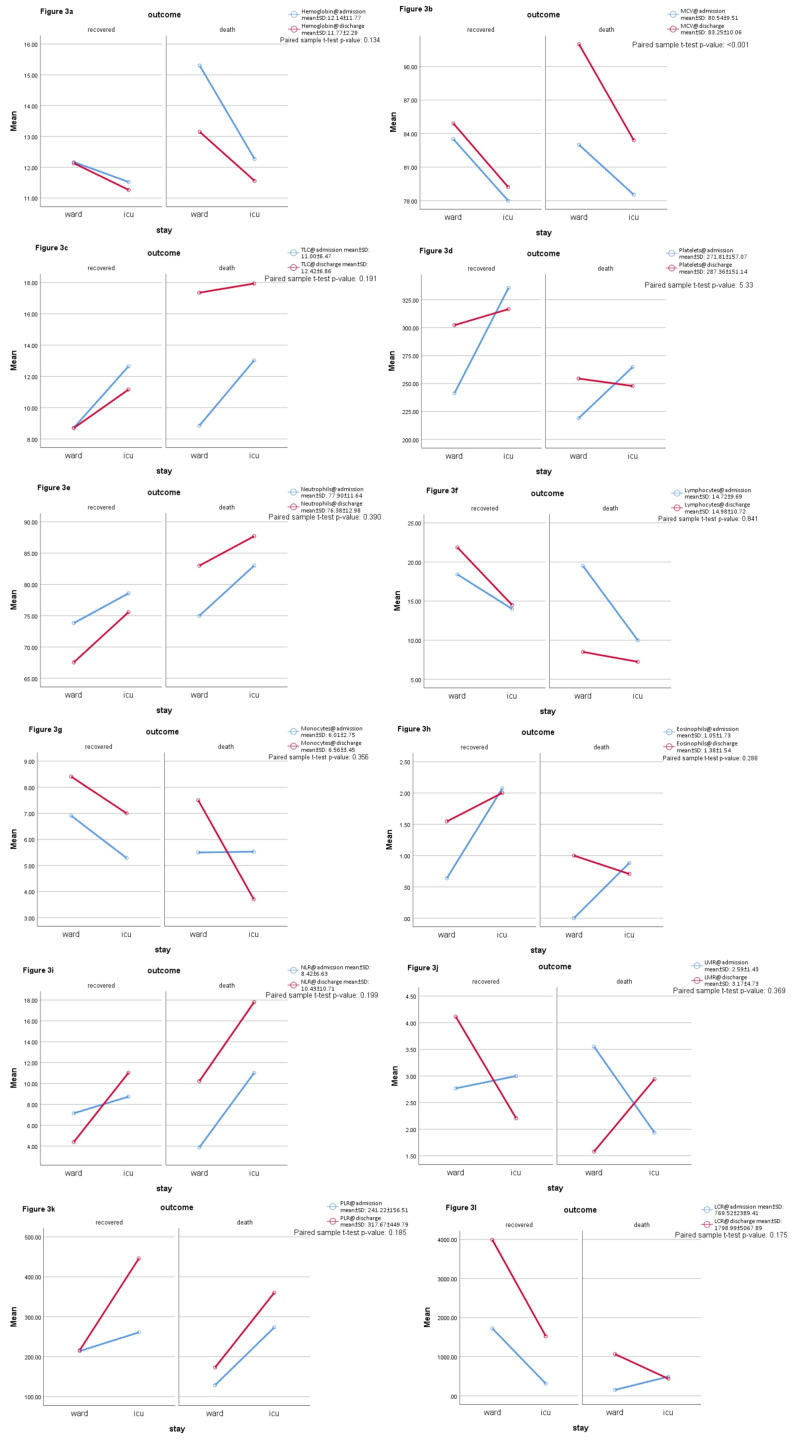
The progress of labs during hospital stay (complete blood picture and hematological indices).

The hemoglobin levels were found to decrease in ICU admission (p = 0.001) as shown in Table 3. Discharging labs also showed decreased hemoglobin in ICU patients and an overall dropping pattern during hospital stay (p = 0.134). The mean cell volume is significantly decreased in ICU patients on admission (p = 0.005) as well as on discharge (p = 0.147) as shown in Table 4 but expired patients showed an increase of mean corpuscular volume (MCV) during hospital stay (p < 0.001). An increased leukocyte count was the most significant finding in ICU (p = 0.001) as well as deceased patients (p = 0.007), with one-half of ICU patients and one-third of deceased patients showed elevated levels above normal range which increased to 84.2% at the time of discharge, while 64.9% recovered patients showed total leukocyte counts (TLC) in the normal range (p < 0.001). An ROC curve demonstrated TLC of 9.30 at 69% sensitivity and 81% positive predictive value (PPV) for ICU admission (AUC: 0.737, p < 0.001) shown in Figure 1, while a TLC of 11.00 has a sensitivity of 63%, a specificity of 79% and 88% PPV in predicting mortality (AUC: 0.727, p=0.001) shown in Figure 2. An improving mean TLC count in recovered patients and an elevated mean TLC in deceased patients can be seen in Figure 3c (p = 0.191). Leucopenia was a feature in only 5 patients, all of them recovered with none had an ICU stay.

**Table 3:** Admitting laboratory investigations of study subjects and their correlation with disease severity. Descriptive statistics are presented as Mean ± standard deviation and Median (interquartile range) respectively. Categorical data is presented as n (%), where n is no. of subjects/total no. of subjects. * indicates independent sample t-test, ** indicates Chi-square test. ^ indicates Mann-Whitney U-test, ” indicates Fisher’s exact test. MCV: Mean corpuscular volume; TLC: Total leukocyte counts; NLR: Neutrophil-to-lymphocyte ratio; LMR: Lymphocyte-to-monocyte ratio; PLR: Platelet-to-lymphocyte ratio; LCR: Lymphocyte-to-C‐reactive protein ratio; ALT: Alanine transaminase; AST: Aspartate aminotransferase; ALP: Alkaline phosphatase; CRP: C-reactive protein; LDH: Lactate dehydrogenase; PCT: Procalcitonin; ESR: Erythrocyte sedimentation rate; BNP: B-type natriuretic peptide.

Correlation of initial laboratory investigations amongst the patients of Covid-19							
#	Laboratory investigation	Ward (n = 67)	ICU (n = 33)	p-value	Recovered (n = 78)	Expired (n = 22)	p-value
Hemogram (complete blood picture)							
1	Hemoglobin (g/dL)	13.29 ± 2.25	11.80 ± 1.94	0.001*	12.90 ± 2.32	12.34 ± 1.98	0.302*
		13.40 (11.80-14.90)	11.90 (10.65-13.30)	0.001^^^	13.10 (11.35-14.60)	12.45 (11.30-13.30)	0.154^^^
2	MCV (fL)	83.45 ± 6.99	77.93 ± 9.62	0.005*	82.48 ± 7.78	78.45 ± 9.64	0.083*
		84.00 (80.00-87.00)	80.00 (73.00-85.00)	0.007^^^	83.50 (78.75-86.25)	80.00 (71.75-85.50)	0.078^^^
3	TLC (10^9^/L)	8.29 ± 4.11	14.25 ± 8.99	0.001*	8.92 ± 5.05	15.10 ± 9.51	0.007*
		7.10 (5.30-10.40)	11.60 (7.90-17.10)	<0.001^^^	7.95 (5.30-10.52)	11.60 (7.35-20.42)	0.001^^^
	>11 x 10^9^/L	n = 11/63 (17.5%)	n = 18/33 (54.5%)	<0.001”	n = 15/74 (20.3%)	n = 14/22 (63.6%)	0.001”
	4-11 x 10^9^/L	n = 47/63 (74.6%)	n = 15/33 (45.5%)		n = 54/74 (73.0%)	n = 8/22 (36.4%)	
	<4 x 10^9^/L	n = 5/63 (7.9%)	n = 0/33 (0.0%)		n = 5/74 (6.8%)	n = 0/22 (0.0%)	
4	Platelets (10^9^/L)	239.31 ± 100.75	295.39 ± 171.42	0.091*	257.87 ± 136.25	261.00 ± 115.97	0.923*
		223.00 (185.00-269.00)	263.00 (183.50-377.00)	0.165^^^	224.00 (184.25-303.25)	249.50 (184.25-335.25)	0.577^^^
	>400 x 10^9^/L	n = 3/63 (4.8%)	n = 7/33 (21.2%)	0.011”	n = 8/74 (10.8%)	n = 2/22 (9.1%)	1.000”
	150-400	n = 53/63 (84.1%)	n = 19/33 (57.6%)		n = 55/74 (74.3%)	n = 17/22 (77.3%)	
	<150 x 10^9^/L	n = 7/63 (11.1%)	n = 7/33 (21.2%)		n = 11/74 (14.9%)	n = 3/22 (13.6%)	
Differential leukocyte counts							
5	Neutrophils (%)	67.38 ± 15.69	81.27 ± 8.68	<0.001*	68.93 ± 15.53	83.00 ± 6.51	<0.001*
		68.00 (58.00-81.00)	83.00 (77.00-88.00)	<0.001^^^	70.50 (58.75-83.25)	82.50 (77.00-88.00)	<0.001^^^
	>75%	n = 20/63 (31.7%)	n = 26/33 (78.8%)	<0.001^**^	n = 27/74 (36.5%)	n = 19/22 (86.4%)	<0.001^**^
	<75%	n = 43/63 (68.3%)	n = 7/33 (21.2%)		n = 47/74 (63.5%)	n = 3/22 (13.6%)	
	Lymphocytes (%)	23.98 ± 13.12	11.33 ± 6.83	<0.001*	22.48 ± 13.01	10.04 ± 5.58	<0.001*
		23.00 (13.00-33.00)	9.00 (6.00-16.50)	<0.001^^^	21.00 (10.00-32.25)	8.50 (5.75-14.50)	<0.001^^^
	>20%	n = 36/63 (57.1%)	n = 3/33 (9.1%)	<0.001^**^	n = 38/74 (51.4%)	n = 1/22 (4.5%)	<0.001^**^
	<20%	n = 27/63 (42.9%)	n = 30/33 (90.9%)		n = 36/74 (48.6%)	n = 21/22 (95.5%)	
	Monocytes (%)	7.09 ± 2.91	5.63 ± 3.02	0.027*	6.86 ± 2.92	5.68 ± 3.21	0.107*
		6.00 (5.00-10.00)	5.00 (3.00-7.00)	0.011^^^	6.00 (5.00-9.00)	5.00 (3.75-6.25)	0.054^^^
	Eosinophils (%)	1.33 ± 1.89	1.36 ± 2.08	0.943*	1.52 ± 2.11	0.72 ± 1.07	0.092*
		1.00 (0.00-2.00)	1.00 (0.00-2.00)	0.715^^^	1.00 (0.00-2.00)	0.00 (0.00-1.00)	0.049^^^
	Basophils (%)	0.11 ± 0.31	0.03 ± 0.17	0.177*	0.09 ± 0.29	0.04 ± 0.21	0.469*
		0.00 (0.00-0.00)	0.00 (0.00-0.00)	0.176^^^	0.00 (0.00-0.00)	0.00 (0.00-0.00)	0.466^^^
Hematological indices							
6	NLR	4.96 ± 5.14	11.19 ± 9.33	0.001*	5.45 ± 5.40	12.65 ± 10.37	0.004*
		3.00 (1.69-6.46)	9.11 (4.60-13.66)	<0.001^^^	3.21 (1.75-8.52)	9.93 (5.45-15.59)	<0.001^^^
	<3	n = 32/63 (50.8%)	n = 1/33 (3.0%)	<0.001^**^	n = 33/74 (44.6%)	n = 0/22 (0.0%)	<0.001^**^
	3-9	n = 20/63 (31.7%)	n = 15/33 (45.5%)		n = 25/74 (33.8%)	n = 10/22 (45.5%)	
	>9	n = 11/63 (17.5%)	n = 17/33 (51.5%)		n = 16/74 (21.6%)	n = 12/22 (54.5%)	
	LMR	3.48 ± 1.69	2.31 ± 1.35	<0.001*	3.40 ± 1.70	1.97 ± 0.98	<0.001*
		3.33 (2.00-4.60)	2.00 (1.22-2.81)	0.001^^^	3.29 (2.00-4.52)	1.80 (1.20-2.80)	<0.001^^^
	PLR	169.81 ± 105.30	271.84 ± 179.47	0.004*	186.38 ± 130.34	267.11 ± 168.05	0.047*
		135.86 (105.41-207.75)	224.76 (142.06-356.92)	0.001^^^	144.24 (105.51-228.75)	220.33 (138.76-353.26)	<0.001^^^
	LCR	2843.20 ± 5533.43	387.39 ± 1293.34	0.002*	2448.18 ± 5153.54	398.42 ± 1536.17	0.070*
		313.24 (83.45-2619.69)	70.25 (41.91-136.61)	0.001^^^	273.26 (77.19-1456.33)	54.13 (32.15-82.41)	<0.001^^^
Renal and electrolytes profile							
7	Urea (mg/dL)	30.04 ± 21.36	58.33 ± 1.94	<0.001*	33.14 ± 24.81	61.47 ± 38.38	0.003*
		25.68 (18.62-35.10)	50.93 (31.24-77.47)	<0.001^^^	26.41 (18.62-36.46)	51.36 (34.18-79.98)	<0.001^^^
	>49 mg/dL	n = 6/67 (9.0%)	n = 17/33 (51.5%)	<0.001^**^	n = 11/78 (14.1%)	n = 12/22 (54.5%)	<0.001^**^
	<49 mg/dL	n = 61/67 (91.0%)	n = 16/33 (48.5%)		n = 67/78 (85.9%)	n = 10/22 (45.5%)	
	Creatinine (mg/dL)	0.91 ± 0.28	1.59 ± 1.18	0.002*	0.99 ± 0.60	1.64 ± 1.09	0.013*
		0.80 (0.72-1.05)	1.17 (0.82-1.97)	<0.001^^^	0.81 (0.72-1.04)	1.25 (1.02-1.86)	<0.001^^^
	>1.3 mg/dL	n = 6/67 (9.0%)	n = 13/33 (39.4%)	<0.001^**^	n = 9/78 (11.5%)	n = 10/22 (45.5%)	0.001”
	<1.3 mg/dL	n = 61/67 (91.0%)	n = 20/33 (60.6%)		n = 69/78 (88.5%)	n = 12/22 (54.5%)	
	Sodium (mEq/L)	137.19 ± 3.97	136.18 ± 6.47	0.337*	137.01 ± 4.68	136.31 ± 5.81	0.562*
		137.00 (135.00-140.00)	137.00 (132.50-140.00)	0.476^^^	137.00 (135.00-140.00)	136.00 (131.75-140.00)	0.323^^^
	Potassium (mEq/L)	4.19 ± 0.59	4.72 ± 0.83	0.002*	4.32 ± 0.69	4.50 ± 0.83	0.321*
		4.10 (3.80-4.50)	4.80 (4.05-5.20)	0.002^^^	4.10 (3.80-4.80)	4.30 (3.87-5.00)	0.428^^^
	Chloride (mEq/L)	102.40 ± 3.97	99.72 ± 7.93	0.075*	102.17 ± 5.35	99.18 ± 6.36	0.053*
		102.00 (100.00-105.00)	99.00 (97.00-104.50)	0.023^^^	102.00 (99.75-105.25)	98.00 (96.75-101.25)	0.002^^^
	Bicarbonate (mEq/L)	19.37 ± 3.37	17.57 ± 4.84	0.062*	19.25 ± 3.55	17.09 ± 4.97	0.067*
		19.00 (18.00-21.00)	18.00 (14.00-21.00)	0.027^^^	19.00 (17.00-21.00)	18.00 (12.75-21.00)	0.040^^^
	<16 mEq/L	n = 9/67 (13.4%)	n = 11/33 (33.3%)	0.019^**^	n = 12/78 (15.4%)	n = 8/22 (36.4%)	0.039”
	>16 mEq/L	n = 58/67 (86.6%)	n = 22/33 (66.7%)		n = 66/78 (84.6%)	n = 14/22 (63.6%)	
Hepatic Function enzymes							
8	Total bilirubin (mg/dL)	0.54 ± 0.32	1.35 ± 2.36	0.122*	0.53 ± 0.31	1.70 ± 2.71	0.105*
		0.46 (0.28-0.75)	0.61 (0.43-0.96)	0.027^^^	0.45 (0.28-0.71)	0.73 (0.50-1.49)	0.002^^^
	>1.0 mg/dL	n = 5/55 (9.1%)	n = 5/22 (22.7%)	0.138”	n = 5/61 (8.2%)	n = 5/16 (31.3%)	0.028”
	Direct (mg/dL)	0.28 ± 0.16	0.89 ± 1.71	0.105*	0.28 ± 0.16	1.13 ± 1.97	0.108*
		0.24 (0.15-0.36)	0.36 (0.24-0.58)	0.005^^^	0.24 (0.15-0.36)	0.45 (0.31-0.81)	0.001^^^
	>0.3 mg/dL	n = 18/55 (32.7%)	n = 15/22 (68.2%)	0.005^**^	n = 20/61 (32.8%)	n = 13/16 (81.3%)	<0.001^**^
	Indirect (mg/dL)	0.23 ± 0.17	0.45 ± 0.66	0.143*	0.23 ± 0.16	0.56 ± 0.75	0.097*
		0.20 (0.12-0.31)	0.26 (0.17-0.39)	0.112^^^	0.20 (0.11-0.31)	0.27 (0.19-0.67)	0.008^^^
	>0.8 mg/dL	n = 1/55 (1.8%)	n = 3/22 (13.6%)	0.068”	n = 1/61 (1.6%)	n = 3/16 (18.8%)	0.027”
	ALT (IU/L)	61.20 ± 154.74	79.72 ± 165.47	0.643*	58.16 ± 147.17	98.25 ± 192.09	0.367*
		32.00 (22.00-46.00)	38.00 (26.50-54.75)	0.248^^^	32.00 (22.00-45.50)	41.50 (31.25-66.75)	0.053^^^
	>45 IU/L	n = 14/55 (25.5%)	n = 8/22 (36.4%)	0.338^**^	n = 15/61 (24.6%)	n = 7/16 (43.8%)	0.212”
	AST (IU/L)	64.29 ± 163.75	97.27 ± 193.78	0.451*	61.63 ± 155.64	119.75 ± 224.72	0.232*
		38.00 (27.00-52.00)	49.50 (41.75-64.25)	0.019^^^	38.00 (27.00-50.50)	49.50 (44.50-66.50)	0.001^^^
	>45 IU/L	n = 18/55 (32.7%)	n = 14/22 (63.6%)	0.013^**^	n = 20/61 (32.8%)	n = 12/16 (75.0%)	0.002^**^
	γGT (IU/L)	72.54 ± 81.90	91.45 ± 90.18	0.377*	71.65 ± 79.19	101.93 ± 100.23	0.202*
		50.00 (25.00-95.00)	73.50 (36.50-116.50)	0.150^^^	49.00 (25.50-94.50)	75.00 (52.50-123.50)	0.056^^^
	>55 IU/L	n = 27/55 (49.1%)	n = 13/22 (59.1%)	0.428^**^	n = 28/61 (45.9%)	n = 12/16 (75.0%)	0.038^**^
	ALP (IU/L)	98.07 ± 51.47	150.68 ± 241.12	0.126*	98.47 ± 48.38	168.87 ± 283.34	0.065*
		88.00 (62.00-118.00)	95.00 (73.00-228.80)	0.346^^^	89.00 (64.50-113.50)	95.50 (70.00-127.50)	0.669^^^
	>135 IU/L	n = 8/55 (14.5%)	n = 3/22 (13.6%)	1.000“	n = 8/61 (13.1%)	n = 3/16 (18.8%)	0.689”
Inflammatory and biochemical markers							
9	CRP (mg/L)	88.35 ± 97.21	177.06 ± 103.68	<0.001*	96.57 ± 100.54	195.27 ± 97.10	<0.001*
		47.60 (10.30-145.60)	173.60 (107.50-279.85)	<0.001^^^	57.95 (14.25-151.20)	174.85 (132.07-292.55)	<0.001^^^
	>10 mg/L	n = 45/59 (76.3%)	n = 31/33 (93.9%)	0.032^**^	n = 55/70 (78.6%)	n = 21/22 (95.5%)	0.105”
10	LDH (U/L)	485.75 ± 354.63	812.90 ± 386.62	0.003*	505.24 ± 342.24	978.67 ± 379.67	0.001*
		396.00 (265.50-575.50)	778.00 (530.03-1002.50)	<0.001^^^	418.00 (275.00-587.50)	885.50 (718.75-1172.25)	<0.001^^^
	>250 U/L	n = 33/40 (82.5%)	n = 21/21 (100.0%)	0.084”	n = 42/49 (85.7%)	n = 12/12 (100.0%)	0.327”
11	Ferritin (ng/mL)	526.38 ± 626.44	1102.66 ± 778.26	0.005*	622.16 ± 684.74	1111.13 ± 795.16	0.058*
		252.07 (265.50-575.50)	1044.30 (530.03-1002.50)	<0.005^^^	295.10 (125.27-953.26)	726.30 (416.93-2000.00)	0.027^^^
	>250 ng/mL for males & >120 ng/mL for females	n = 22/44 (50.0%)	n = 18/22 (81.8%)	0.013^**^	n = 29/53 (54.7%)	n = 11/13 (84.6%)	0.048^**^
12	PCT (ng/mL)	0.68 ± 1.30	4.69 ± 15.09	0.463*	4.47 ± 17.31	2.96 ± 6.05	0.742*
		0.09 (0.03-0.85)	0.22 (0.05-1.25)	0.542^^^	0.06 (0.03-0.55)	0.47 (0.11-2.07)	0.023^^^
	>0.5 ng/mL	n = 2/8 (25.0%)	n = 10/28 (35.7%)	0.691”	n = 5/20 (25.0%)	n = 7/16 (43.8%)	0.236^**^
13	D-dimer (mcg/mL)	1.49 ± 2.13	3.45 ± 2.76	0.175*	2.89 ± 3.33	2.32 ± 1.89	0.701*
		0.60 (0.43-2.31)	3.18 (1.28-4.48)	0.093^^^	1.15 (0.48-4.76)	1.92 (0.61-3.30)	0.848^^^
	>0.5 mcg/mL	n = 4/6 (66.7%)	n = 8/8 (100.0%)	0.165”	n = 5/7 (71.4%)	n = 7/7 (100.0%)	0.462”
14	ESR (mm/hour)	75.00 ± 38.18	77.14 ± 43.75	0.952*	83.20 ± 30.21	68.50 ± 54.21	0.620*
		75.00 (48.00-102.00)	78.00 (24.00-124.00)	0.769^^^	78.00 (56.00-113.00)	65.00 (21.00-119.50)	0.712^^^
	>20 mm/hour	n = 2/2 (100.0%)	n = 6/7 (85.7%)	1.000”	n = 5/5 (100.0%)	n = 3/4 (75.0%)	0.444”
15	Trop I (pg/mL)	108.01 ± 134.71	100.99 ± 161.48	0.918*	94.71 ± 134.57	110.90 ± 165.65	0.807*
		65.40 (3.20-198.72)	19.25 (10.87-127.87)	0.539^^^	18.45 (3.20-228.15)	20.20 (10.57-127.72)	0.428^^^
	>34.2 pg/mL for males & >15.6 pg/mL for females	n = 4/8 (50.0%)	n = 5/14 (35.7%)	0.662”	n = 4/10 (40.0%)	n = 5/12 (41.7%)	1.000”
16	Pro-BNP (pg/mL)	5661.80 ± 7697.54	24891.07 ± 64635.74	0.485*	23128.95 ± 64690.99	8892.35 ± 13217.30	0.607*
		1406.00 (80.30-15308.95)	1004.80 (295.00-8991.70)	0.482^^^	316.50 (90.30-15108.80)	4279.05 (455.72-15493.77)	0.269^^^
	>125 pg/mL	n = 4/6 (66.7%)	n = 10/11 (90.9%)	0.515”	n = 8/11 (72.7%)	n = 6/6 (100.0%)	0.515”

**Table 4:** Discharging laboratory investigations of study subjects and their correlation with disease severity. Descriptive statistics are presented as Mean ± standard deviation and Median (interquartile range) respectively. Categorical data is presented as n (%), where n = no. of subjects/total no. of subjects. * indicates independent sample t-test, ** indicates Chi-square test. ^ indicates Mann-Whitney U-test, ” indicates Fisher’s exact test. MCV: Mean corpuscular volume; TLC: Total leukocyte counts; NLR: Neutrophil-to-lymphocyte ratio; LMR: Lymphocyte-to-monocyte ratio; PLR: Platelet-to-lymphocyte ratio; LCR: Lymphocyte-to-C‐reactive protein ratio; ALT: Alanine transaminase; AST: Aspartate aminotransferase; ALP: Alkaline phosphatase; CRP: C-reactive protein; LDH: Lactate dehydrogenase; ESR: Erythrocyte sedimentation rate; BNP: B-type natriuretic peptide.

#	Laboratory investigation	Ward (n = 67)	ICU (n = 33)	p-value	Recovery (n = 78)	Death (n = 22)	p-value
1	Hemoglobin	12.21 ± 2.36	11.42 ± 2.20	0.208*	11.79 ± 2.41	11.72 ± 2.10	0.918*
		11.90 (10.92-14.12)	11.00 (9.90-12.80)	0.194^^^	11.45 (9.92-13.45)	11.40 (10.20-13.90)	0.951^^^
2	MCV	85.50 ± 9.20	81.51 ± 10.50	0.147*	82.69 ± 9.29	84.31 ± 11.59	0.575*
		86.00 (82.00-90.00)	80.00 (75.00-90.00)	0.079^^^	84.00 (78.00-87.75)	83.00 (77.00-92.00)	0.620^^^
3	TLC	9.38 ± 4.09	14.87 ± 7.69	0.001*	9.62 ± 3.99	17.87 ± 8.04	<0.001*
		9.30 (5.95-11.50)	13.60 (9.30-19.60)	0.004^^^	9.40 (6.15-11.90)	15.30 (13.40-23.20)	<0.001^^^
	>11 x 10^9^/L	n = 7/25 (28.0%)	n = 21/31 (67.8%)	0.006”	n = 12/37 (32.4%)	n = 16/19 (84.2%)	<0.001”
	4-11 x 10^9^/L	n = 17/25 (68.0%)	n = 9/31 (29.0%)		n = 24/37 (64.9%)	n = 2/19 (10.5%)	
	<4 x 10^9^/L	n = 1/25 (4.0%)	n = 1/31 (3.2%)		n = 1/37 (2.7%)	n = 1/19 (5.3%)	
4	Platelet	298.29 ± 121.72	278.90 ± 172.01	0.641*	307.86 ± 133.36	248.52 ± 177.51	0.168*
		284.00 (179.00-406.25)	224.00 (154.00-397.00)	0.245^^^	272.00 (213.50-412.50)	171.50 (131.00-346.00)	0.045^^^
	>400 x 10^9^/L	n = 7/24 (29.2%)	n = 7/31 (22.6%)	0.189”	n = 11/36 (30.6%)	n = 3/19 (15.8%)	0.004”
	150-400	n = 16/24 (66.7%)	n = 17/31 (54.8%)		n = 24/36 (66.7%)	n = 9/19 (47.4%)	
	<150 x 10^9^/L	n = 1/24 (4.2%)	n = 7/31 (22.6%)		n = 1/36 (2.8%)	n = 7/19 (36.8%)	
5	Neutrophil	68.83 ± 11.91	82.22 ± 10.66	<0.001*	70.66 ± 12.05	87.21 ± 5.84	<0.001*
		71.00 (61.25-76.75)	85.00 (76.00-92.00)	<0.001^^^	72.00 (62.25-79.25)	88.00 (83.00-92.00)	<0.001^^^
	>75%	n = 7/24 (29.2%)	n = 24/31 (77.4%)	<0.001^**^	n = 12/36 (33.3%)	n = 19/19 (100.0%)	<0.001^**^
6	Lymphocyte	20.75 ± 10.55	10.51 ± 7.92	<0.001*	19.00 ± 10.46	7.36 ± 4.41	<0.001*
		20.50 (11.50-27.50)	8.00 (4.00-15.00)	<0.001^^^	17.00 (11.25-25.75)	7.00 (4.00-10.00)	<0.001^^^
	<20%	n = 12/24 (50.0%)	n = 27/31 (87.1%)	0.003^**^	n = 20/36 (55.6%)	n = 19/19 (100.0%)	0.001^**^
7	Monocyte	8.33 ± 3.49	5.19 ± 2.77	0.001*	7.86 ± 3.25	4.10 ± 2.35	<0.001*
		8.00 (6.00-10.75)	5.00 (3.00-7.00)	0.001^^^	8.00 (6.00-9.75)	4.00 (3.00-5.00)	<0.001^^^
8	Eosinophil	1.50 ± 1.38	1.29 ± 1.67	0.622*	1.72 ± 1.64	0.73 ± 1.09	0.011*
		1.50 (0.00-2.75)	1.00 (0.00-2.00)	0.356^^^	1.00 (0.00-3.00)	0.00 (0.00-2.00)	0.022^^^
9	Basophil	0.16 ± 0.38	0.12 ± 0.34	0.701*	0.19 ± 0.40	0.05 ± 0.22	0.162*
		0.00 (0.00-0.00)	0.00 (0.00-0.00)	0.697^^^	0.00 (0.00-0.00)	0.00 (0.00-0.00)	0.160^^^
10	NLR	4.87 ± 3.78	14.74 ± 12.32	<0.001*	6.97 ± 8.81	17.00 ± 11.14	0.002*
		3.63 (2.24-6.85)	10.87 (5.20-22.50)	<0.001^^^	4.34 (2.39-6.93)	12.85 (8.50-22.75)	<0.001^^^
	<3	n = 9/24 (37.5%)	n = 3/31 (9.7%)	0.001^**^	n = 12/36 (33.3%)	n = 0/19 (0.0%)	<0.001”
	3-9	n = 13/24 (54.2%)	n = 11/31 (35.5%)		n = 19/36 (52.8%)	n = 5/19 (26.3%)	
	>9	n = 2/24 (8.3%)	n = 17/31 (54.8%)		n = 5/36 (13.9%)	n = 14/19 (73.7%)	
11	LMR	3.90 ± 6.20	2.60 ± 3.16	0.319*	3.37 ± 5.14	2.79 ± 3.94	0.672*
		2.66 (2.00-3.87)	1.66 (0.83-3.50)	0.140^^^	2.44 (1.75-3.66)	1.60 (0.80-3.75)	0.144^^^
12	PLR	212.68 ± 196.08	398.95 ± 564.84	0.129*	305.63 ± 466.06	340.48 ± 428.64	0.788*
		153.88 (120.02-226.14)	186.40 (109.89-334.48)	0.359^^^	155.01 (125.18-291.45)	186.40 (107.75-238.82)	0.986^^^
13	LCR	3598.54 ± 8414.28	928.25 ± 1755.74	0.094*	2710.88 ± 6477.16	503.16 ± 767.69	0.148*
		1106.21 (273.85-2971.42)	201.76 (91.74-760.28)	0.021^^^	623.97 (259.31-2380.00)	194.03 (91.74-598.47)	0.022^^^
14	Urea	40.65 ± 38.80	117.46 ± 86.51	<0.001*	38.87 ± 27.22	166.75 ± 76.56	<0.001*
		28.40 (20.44-40.92)	106.25 (31.99-168.04)	<0.001^^^	28.99 (24.07-40.92)	151.30 (122.72-201.42)	<0.001^^^
	>49 mg/dL	n = 5/24 (20.8%)	n = 22/32 (68.8%)	<0.001^**^	n = 8/36 (22.2%)	n = 19/20 (95.0%)	<0.001^**^
15	Creatinine	1.15 ± 1.04	2.64 ± 2.53	0.004*	1.08 ± 1.00	3.65 ± 2.66	<0.001*
		0.86 (0.76-1.04)	1.60 (0.87-3.60)	0.002^^^	0.87 (0.76-1.04)	3.05 (1.60-4.98)	<0.001^^^
	>1.3 mg/dL	n = 2/24 (8.3%)	n = 18/32 (56.3%)	<0.001^**^	n = 3/36 (8.3%)	n = 17/20 (85.0%)	<0.001^**^
16	Sodium	137.75 ± 5.69	144.61 ± 12.05	0.008*	136.28 ± 3.26	150.95 ± 11.79	<0.001*
		137.00 (135.00-139.75)	141.00 (136.00-151.00)	0.048^^^	136.00 (134.00-138.00)	150.50 (143.50-157.00)	<0.001^^^
17	Potassium	4.04 ± 0.67	4.61 ± 1.26	0.035*	4.03 ± 0.70	4.95 ± 1.36	0.009*
		4.05 (3.55-4.47)	4.50 (3.60-5.50)	0.082^^^	4.10 (3.50-4.50)	5.10 (3.70-5.65)	0.008^^^
18	Chloride	101.41 ± 5.93	104.32 ± 11.26	0.257*	99.11 ± 5.53	109.95 ± 10.74	<0.001*
		103.00 (97.25-105.75)	102.00 (98.00-111.00)	0.586^^^	99.00 (97.00-103.00)	109.50 (102.25-115.50)	<0.001^^^
19	Bicarbonate	21.29 ± 3.34	20.41 ± 7.09	0.581*	22.51 ± 4.93	17.80 ± 5.92	0.005*
		20.00 (19.25-23.00)	19.00 (15.00-24.00)	0.233^^^	22.00 (19.00-24.00)	18.50 (14.25-20.00)	0.002^^^
	<16 mEq/L	n = 1/24 (4.2%)	n = 9/31 (29.0%)	0.031”	n = 2/35 (5.7%)	n = 8/20 (40.0%)	0.003”
20	Total bilirubin	0.44 ± 0.56	1.33 ± 0.51	0.094*	0.79 ± 0.68	1.08 ± 0.40	0.617*
		0.43 (0.40-0.47)	1.37 (0.80-1.59)	0.050^^^	0.47 (0.40-1.49)	1.08 (0.80-1.37)	0.355^^^
	>1.0 mg/dL	n = 0/3 (0.0%)	n = 2/3 (66.7%)	0.400”	n = 1/4 (25.0%)	n = 1/2 (50.0%)	1.000”
21	Direct bilirubin	0.28 ± 0.30	0.72 ± 0.35	0.099*	0.43 ± 0.30	0.64 ± 0.45	0.530*
		0.28 (0.26-0.30)	0.89 (0.60-0.93)	0.077^^^	0.30 (0.26-0.74)	0.64 (0.32-0.97)	0.240^^^
	>0.3 mg/dL	n = 1/3 (33.3%)	n = 3/3 (100.0%)	0.400”	n = 2/4 (50.0%)	n = 2/2 (100.0%)	0.467”
22	Indirect bilirubin	0.16 ± 0.02	0.60 ± 0.28	0.055*	0.35 ± 0.38	0.44 ± 0.05	0.778*
		0.15 (0.14-0.17)	0.48 (0.40-0.70)	0.050^^^	0.17 (0.14-0.74)	0.44 (0.40-0.48)	0.355^^^
	>0.8 mg/dL	n = 1/3 (33.3%)	n = 1/3 (33.3%)	1.000”	n = 1/4 (25.0%)	n = 0/2 (0.0%)	1.000”
23	ALT	46.33 ± 22.94	150.00 ± 156.61	0.320*	116.25 ± 141.08	62.00 ± 50.91	0.642*
		57.00 (38.50-59.50)	98.00 (26.00-212.00)	0.275^^^	59.50 (29.25-260.00)	62.00 (26.00-98.00)	1.000^^^
	>45 IU/L	n = 2/3 (66.7%)	n = 2/3 (66.7%)	1.000”	n = 3/4 (75.0%)	n = 1/2 (50.0%)	1.000”
24	AST	41.33 ± 20.42	131.33 ± 95.97	0.187*	48.75 ± 22.32	161.50 ± 113.84	0.096*
		50.00 (34.00-53.00)	81.00 (71.00-161.50)	0.050^^^	53.00 (26.00-67.25)	161.50 (81.00-242.00)	0.064^^^
	>45 IU/L	n = 2/3 (66.7%)	n = 3/3 (100.0%)	1.000”	n = 3/4 (75.0%)	n = 2/20 (100.0%)	1.000”
25	γGT	47.00 ± 33.00	89.33 ± 42.19	0.243*	69.75 ± 52.87	65.00 ± 2.82	0.910*
		47.00 (14.00-63.50)	67.00 (63.00-102.50)	0.275^^^	63.50 (22.25-123.50)	65.00 (63.00-67.00)	1.000^^^
	>55 IU/L	n = 1/3 (33.0%)	n = 3/3 (100.0%)	0.400”	n = 2/4 (50.0%)	n = 2/2 (100.0%)	0.467”
26	ALP	152.00 ± 89.11	115.33 ± 11.01	0.518*	141.50 ± 75.72	118.00 ± 14.14	0.702*
		181.00 (52.00-202.00)	110.00 (108.00-119.00)	0.513^^^	145.50 (66.50-212.50)	118.00 (108.00-128.00)	0.643^^^
	>135 IU/L	n = 2/3 (66.7%)	n = 0/3 (0.0%)	0.400”	n = 2/4 (50.0%)	n = 0/2 (0.0%)	0.467”
27	CRP	38.01 ± 42.18	65.54 ± 69.04	0.133*	45.88 ± 51.05	70.50 ± 74.26	0.175*
		22.20 (6.50-57.85)	42.40 (14.10-92.90)	0.138^^^	25.60 (8.15-67.60)	42.50 (22.60-110.50)	0.107^^^
	>10.0 mg/dL	n = 12/18 (66.7%)	n = 25/31 (80.6%)	0.316”	n = 20/30 (66.7%)	n = 17/19 (89.5%)	0.095”
28	LDH	391.00 ± 79.18	704.71 ± 413.76	0.243*	500.25 ± 241.48	1052.00 ± 592.55	0.053*
		360.00 (332.00-420.50)	633.00 (337.00-995.00)	0.210^^^	420.50 (333.25-632.25)	1052.00 (633.00-1471.00)	0.117^^^
	>250 U/L	n = 3/3 (100.0%)	n = 7/7 (100.0%)	1.000”	n = 8/8 (100.0%)	n = 2/2 (100.0%)	1.000”
29	Ferritin	865.70 ± 985.07	1737.47 ± 371.26	0.334*	865.70 ± 985.07	1737.47 ± 371.26	0.334*
		372.00 (225.11-1186.00)	1737.47 (1474.95-2000.00)	0.374^^^	372.00 (225.11-1186.00)	1737.47 (1474.94-2000.00)	0.374^^^
	>250 ng/mL (males) >120 ng/mL (females)	n = 2/3 (66.7%)	n = 2/2 (100.0%)	1.000”	n = 2/3 (66.7%)	n = 2/2 (100.0%)	1.000”
30	Procalcitonin	0.05 ± 0.04	6.84 ± 17.41	0.599*	11.44 ± 26.97	3.10 ± 6.48	0.334*
		0.05 (0.02-0.08)	0.52 (0.16-3.54)	0.086^^^	0.21 (0.02-17.88)	0.52 (0.08-3.54)	0.481^^^
	>0.5 ng/mL	n = 0/2 (0.0%)	n = 8/15 (53.3%)	0.471”	n = 2/6 (33.3%)	n = 6/11 (54.5%)	0.620”
31	D-dimer	3.14 ± 3.76	1.98 ± 0.53	0.708*	1.98 ± 0.53	3.14 ± 3.76	0.708*
		3.14 (0.48-5.81)	1.98 (1.61-2.36)	1.000^^^	1.98 (1.61-2.36)	3.14 (0.48-5.81)	0.100^^^
	>0.5 mcg/mL	n = 1/2 (50.0%)	n = 2/2 (100.0%)	1.000”	n = 2/2 (100.0%)	n = 1/2 (50.0%)	1.000”
32	ESR	92.00 ± 19.79	68.50 ± 28.99	0.444*	63.00 ± 21.21	97.50 ± 12.02	0.183*
		92.00 (78.00-106.00)	68.50 (48.00-89.00)	0.439^^^	63.00 (48.00-78.00)	97.50 (89.00-106.00)	0.121^^^
	>20 mm/hour	n = 2/2 (100.0%)	n = 2/2 (100.0%)	1.000”	n = 2/2 (100.0%)	n = 2/2 (100.0%)	1.000”
33	Trop I	95.20 ± 129.96	135.26 ± 155.70	0.757*	106.46 ± 93.94	136.52 ± 174.05	0.796*
		95.20 (3.30-187.10)	104.80 (11.90-219.40)	0.739^^^	129.00 (3.30-158.05)	80.60 (11.20-289.80)	0.881^^^
	>34 pg/mL (males) >16 pg/mL (females)	n = 1/2 (50.0%)	n = 4/6 (66.7%)	1.000”	n = 2/3 (66.7%)	n = 3/5 (60.0%)	1.000”
34	Pro-BNP	7448.15 ± 6742.75	843.45 ± 228.18	0.300*	6449.05 ± 8155.69	1842.55 ± 1184.75	0.512*
		7448.15 (2680.30-12216.00)	843.45 (682.10-1004.80)	0.121^^^	6449.05 (682.10-12216.00)	1842.55 (1004.80-2680.30)	1.000^^^
	>125 pg/mL	n = 2/2 (100.0%)	n = 2/2 (100.0%)	1.000”	n = 2/2 (100.0%)	n = 2/2 (100.0%)	1.000”

All severe patients showed comparatively increased platelet counts on admission (p = 0.091), although the majority of them had normal range platelet counts, with only 10 patients had thrombocytosis (seven in ICU), and 14 patients had thrombocytopenia (seven in ICU, seven in the ward). Table 4 shows that at discharge, those with thrombocytosis remain static while the frequency of thrombocytopenia was 36% in dying patients (p = 0.004), with only one recovered patient had thrombocytopenia and 30.6% had thrombocytosis depicted an overall incremental response of platelets in survivors and a slight decremental in non-survivors (Figure 3d).

2) Differential Leukocyte Counts

Neutrophil and lymphocyte are the major predictors of disease severity and mortality in COVID-19, with elevated neutrophils a feature of 78% ICU admissions, and 86% mortalities, while lymphocytopenia was present in 91% ICU admissions and 95% mortalities (p < 0.001). At discharge, only one-third recovered had neutrophilia (p < 0.001), and one-half had lymphocytopenia as compared to 100% non-survivors having both (p = 0.001). There was a decreased monocyte count in severe patients both at admission and discharge while eosinophils and basophils remain static. An ROC curve depicted 74.50% neutrophils cut-off for ICU admission (AUC: 0.773, p < 0.001) at a sensitivity of 81.8% and PPV of 87.5%, while cut-off for mortality being 72.50% (AUC: 0.775, p < 0.001) with 95% sensitivity and 97% PPV. Lymphocyte showed an inverse related to disease severity with AUC of 0.203 for ICU stay and AUC of 0.195 for non-survival predicted, as shown in Table 5. Figure 3 demonstrates an overall progression of all CBC indices during the hospital stay with a paired sample comparison of mean values.

**Table 5:** Receiver operating characteristic curve findings of the COVID-19 patients. PPV: Positive predictive value; NPV: Negative predictive value; ICU: Intensive care unit; AUC: Area under curve; S.E: Standard error of mean; ROC: Receiver operating characteristic; MCV: Mean corpuscular volume; TLC: Total leukocyte counts; NLR: Neutrophil-to-lymphocyte ratio; LMR: Lymphocyte-to-monocyte ratio; PLR: Platelet-to-lymphocyte ratio; LCR: Lymphocyte-to-C‐reactive protein ratio; ALT: Alanine transaminase; AST: Aspartate aminotransferase; ALP: Alkaline phosphatase; CRP: C-reactive protein; LDH: Lactate dehydrogenase.

ROC statistics of the Covid-19 patients for ICU admissions and deaths (severity of the disease)										
Variable state			AUC	S.E	95% confidence interval	Sensitivity	Specificity	PPV	NPV	p-value
Hemogram (complete blood picture)										
1	Hemoglobin	ICU (cut off: 12.85)	0.301	0.054	0.196-0.407	36.4%	42.9%	56.3%	25.0%	0.001
		Death (cut off: 11.45)	0.400	0.063	0.276-0.523	77.3%	25.7%	79.2%	23.6%	0.154
	MCV	ICU (cut off: 84.50)	0.333	0.061	0.213-0.453	30.3%	55.6%	60.3%	26.3%	0.007
		Death (cut off: 84.50)	0.376	0.073	0.233-0.519	31.8%	58.1%	74.1%	18.4%	0.078
	TLC	ICU (cutoff: 9.30)	0.737	0.055	0.629-0.845	69.7%	71.4%	81.8%	56.1%	<0.001
		Death (cutoff: 11.00)	0.727	0.063	0.603-0.850	63.6%	79.7%	88.1%	48.3%	0.001
	Platelet	ICU (cut off: 230.00)	0.587	0.067	0.455-0.718	60.6%	58.7%	74.0%	43.5%	0.165
		Death (cut off: 230.00)	0.539	0.071	0.400-0.678	59.1%	55.4%	82.0%	28.3%	0.577
Differential leukocyte counts										
2	Neutrophil	ICU (cutoff: 74.50)	0.773	0.048	0.680-0.867	81.8%	66.7%	87.5%	56.3%	<0.001
		Death (cutoff: 72.50)	0.775	0.047	0.683-0.867	95.5%	58.1%	97.7%	40.4%	<0.001
	Lymphocyte	ICU (cutoff: 7.50)	0.203	0.045	0.115-0.290	69.7%	11.1%	41.2%	29.1%	<0.001
		Death (cutoff: 11.50)	0.195	0.045	0.106-0.284	40.9%	29.7%	62.9%	14.8%	<0.001
	Monocyte	ICU (cut off: 6.50)	0.344	0.060	0.226-0.462	30.3%	58.7%	61.7%	27.8%	0.012
		Death (cut off: 4.50)	0.366	0.068	0.233-0.499	72.7%	23.0%	73.9%	21.9%	0.057
	Eosinophil	ICU (cut off: 0.50)	0.478	0.063	0.354-0.603	54.5%	38.1%	61.5%	31.6%	0.728
		Death (cut off: 0.50)	0.368	0.067	0.238-0.499	40.9%	35.1%	66.7%	15.8%	0.062
	Basophil	ICU (cutoff: 0.50)	0.460	0.061	0.340-0.579	3.0%	88.9%	63.6%	12.5%	0.579
		Death (cutoff: 655.00)	0.475	0.069	0.341-0.610	4.5%	90.5%	76.1%	12.5%	0.727
Hematological indices										
3	NLR	ICU (cut off: 3.71)	0.799	0.046	0.709-0.889	87.9%	62.1%	90.7%	54.7%	<0.001
		Death (cut off: 4.16)	0.806	0.046	0.716-0.895	90.9%	62.2%	95.8%	41.7%	<0.001
	LMR	ICU (cutoff: 2.38)	0.279	0.061	0.169-0.389	48.5%	30.2%	52.8%	26.7%	<0.001
		Death (cutoff: 1.37)	0.237	0.053	0.133-0.342	72.7%	13.5%	62.5%	20.0%	<0.001
	PLR	ICU (cutoff: 153.65)	0.696	0.056	0.576-0.816	72.7%	65.1%	82.0%	52.2%	0.002
		Death (cutoff: 153.65)	0.671	0.070	0.535-0.808	72.7%	59.5%	88.0%	34.8%	0.016
	LCR	ICU (cut off: 38.65)	0.258	0.053	0.154-0.361	78.8%	15.5%	56.3%	34.7%	<0.001
		Death (cut off:38.65)	0.225	0.053	0.120-0.330	72.7%	14.5%	62.5%	21.3%	<0.001
Renal and electrolytes profile										
4	Urea	ICU (cut off: 28.78)	0.795	0.050	0.697-0.893	84.8%	61.2%	89.1%	51.9%	<0.001
		Death (cut off: 33.27)	0.784	0.059	0.668-0.900	81.8%	66.7%	92.9%	40.9%	<0.001
	Creatinine	ICU (cutoff: 0.79)	0.727	0.058	0.614-0.841	84.8%	49.3%	86.8%	45.2%	<0.001
		Death (cutoff: 0.95)	0.808	0.054	0.702-0.914	86.4%	65.4%	94.4%	41.3%	<0.001
	Sodium	ICU (cut off: 137.50)	0.456	0.065	0.329-0.583	45.5%	53.7%	66.7%	32.6%	0.477
		Death (cut off: 139.50)	0.431	0.076	0.282-0.580	31.8%	66.7%	77.6%	21.2%	0.324
	Potassium	ICU (cutoff: 4.25)	0.690	0.061	0.571-0.808	66.7%	64.2%	79.6%	47.8%	0.002
		Death (cutoff: 4.25)	0.555	0.071	0.416-0.685	54.5%	56.4%	81.5%	26.1%	0.429
	Chloride	ICU (cutoff: 103.50)	0.360	0.066	0.230-0.490	27.3%	56.7%	61.3%	23.7%	0.023
		Death (cutoff: 105.50)	0.288	0.071	0.149-0.428	18.2%	75.6%	76.6%	17.4%	0.002
	Bicarbonate	ICU (cut off: 17.50)	0.364	0.063	0.240-0.489	54.5%	22.4%	50.0%	25.7%	0.028
		Death (cut off: 17.50)	0.357	0.073	0.214-0.500	54.5%	25.6%	66.7%	17.1%	0.041
Hepatic function enzymes										
5	Bilirubin	ICU (cut off: 0.50)	0.662	0.066	0.534-0.790	68.2%	60.0%	82.5%	40.5%	0.027
		Death (cut off: 0.47)	0.752	0.064	0.627-0.877	87.5%	52.5%	94.1%	32.6%	0.002
	ALT	ICU (cut off: 24.50)	0.585	0.070	0.448-0.721	86.4%	34.5%	86.4%	34.5%	0.248
		Death (cut off: 26.50)	0.658	0.068	0.524-0.792	87.5%	42.6%	92.9%	28.6%	0.053
	AST	ICU (cutoff: 42.50)	0.672	0.068	0.538-0.806	77.3%	65.5%	87.8%	47.2%	0.019
		Death (cutoff: 42.50)	0.767	0.056	0.658-0.877	87.5%	63.9%	95.1%	38.9%	0.001
	γGT	ICU (cut off: 47.00)	0.605	0.067	0.474-0.737	72.7%	47.3%	81.3%	35.6%	0.151
		Death (cut off: 50.50)	0.535	0.088	0.528-0.785	81.3%	52.5%	91.4%	31.0%	0.056
	ALP	ICU (cutoff: 72.50)	0.569	0.069	0.434-0.704	81.8%	40.0%	84.6%	35.3%	0.346
		Death (cutoff: 72.50)	0.656	0.066	0.363-0.707	75.0%	36.1%	84.6%	23.5%	0.669
Inflammatory biomarkers										
6	CRP	ICU (cutoff: 103.60)	0.806	0.057	0.695-0.917	81.0%	73.0%	85.1%	57.8%	<0.001
		Death (cutoff: 123.65)	0.823	0.057	0.712-0.934	91.7%	71.7%	92.3%	45.0%	0.001
	LDH	ICU (cut off: 513.00)	0.793	0.062	0.671-0.915	85.7%	70.3%	90.3%	60.0%	<0.001
		Death (cut off: 574.50)	0.877	0.045	0.788-0.966	91.7%	73.9%	97.3%	45.8%	<0.001
	Ferritin	ICU (cut off: 338.28)	0.687	0.078	0.534-0.840	81.0%	56.8%	86.7%	50.0%	0.019
		Death (cut off: 388.51)	0.658	0.084	0.493-0.822	83.3%	54.3%	93.9%	33.3%	0.095

3) Hematological Indices

The neutrophil-to-lymphocyte ratio (NLR) was found the most sensitive hematological marker with an AUC of 0.799 for ICU stay at 88% sensitivity and 90% PPV, while a value of 4.16 predicted mortality at a sensitivity of 91% and PPV of 96% (AUC: 0.806, p < 0.001). Around 90% of ICU patients along with non-survivors had NLR > 3 and one-half of them had NLR > 9 at admission. At discharge, neither of non-surviving patients had NLR < 3 as opposed to 73.7% having NLR > 9, while amongst the recovered patients, one-third had NLR < 3, one-half lying between NLR 3-9, and only 13% had NLR > 9 (p < 0.001). Non-severe patients had decreased NLR response at recovery as shown in Figure 3i, as opposed to incremental response in deceased patients (p = 0.199).

Platelet-to-lymphocyte ratio (PLR) was elevated more in ICU (p = 0.004), and deceased patients (p = 0.047), with an AUC of 0.696 and 0.671 respectively at 153.65 cut-off with 72% sensitivity. ICU recoveries had a significantly increased PLR as compared to ward recoveries as shown in Figure 3k (p = 0.185). Lymphocyte-to-monocyte ratio (LMR) and lymphocyte-to-C‐reactive protein ratio (LCR) were inversely related to disease severity. LMR is significantly lower in ICU and deceased patients at admission (p < 0.001), while LCR of discharging patients was decreased in ICU and non-surviving patients (p = 0.148).

4) Renal and Electrolyte Profile

Renal profile was deranged in almost one-half of ICU admissions with a further increase to two-thirds during the hospital stay, with around 90% of deaths reported with increased urea and creatinine levels. Twenty percent of the recovered patients also had an acute renal injury during the hospital stay. An AUC of 0.795 has demonstrated ICU admission for urea levels greater than 28.78 with a sensitivity of 85% and PPV of 90%, and for levels greater than 33.27 (AUC: 0.784) for deceased patients. Serum creatinine greater than 0.79 mg/dL has also been predicted for ICU stay (AUC: 0.727), with 85% sensitivity and 88% PPV, while for mortality an AUC of 0.808 at levels greater than 0.95 mg/dL has been demonstrated at 86% sensitivity and 94% PPV.

**Figure 4:**
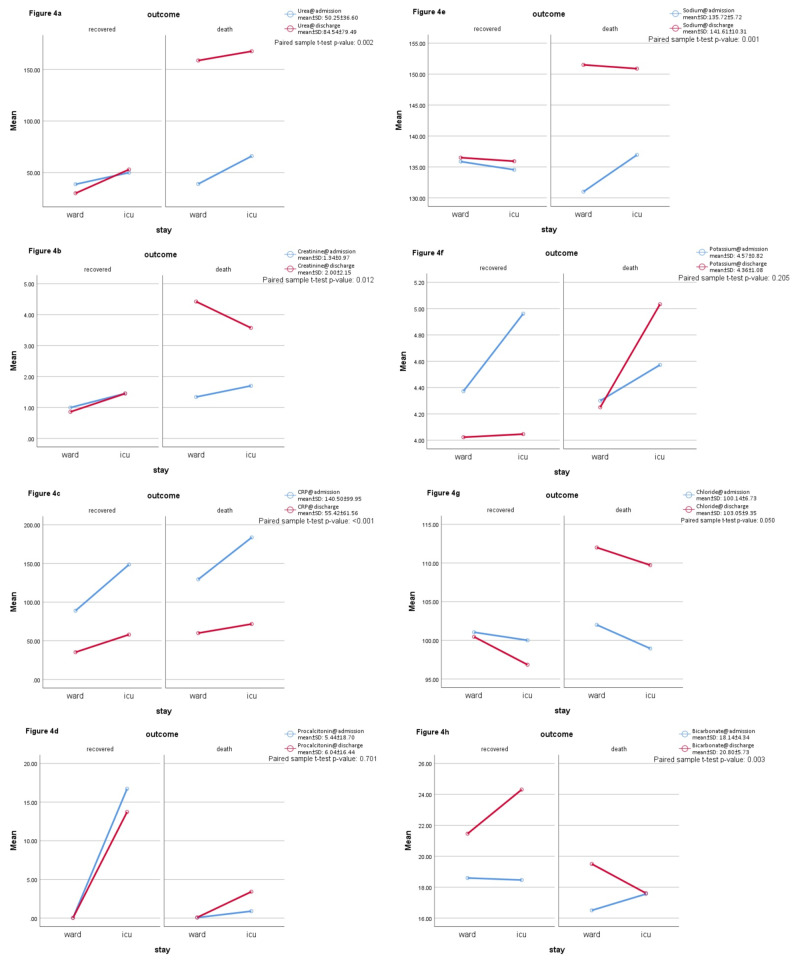
The progress of labs during hospital stay (inflammatory and biochemical markers).

Serum sodium and chloride levels were in the normal range for most of ICU admissions and recoveries, but deceased patients showed an in-hospital increase in both levels (p < 0.001). Serum potassium has slightly elevated levels at admission as well as at discharge for ICU stay and mortalities but recovered patients showed a decreased potassium at discharge although usually within normal ranges (Figure 4f). A serum bicarbonate level of <16 mEq/L was present in one-third of ICU admissions (p = 0.019) and at the discharge of deceased patients (p = 0.003). Figure 4h also shows increased bicarbonate of recovering patients at discharge.

5) Hepatic Function Enzymes

The most frequently deranged liver enzyme was found to be a gamma-glutamyl transferase, in around 40 patients followed by elevated aspartate aminotransferase in 32 patients; both were found more frequently deranged in ICU admissions and as well as non-surviving patients. The least affected enzyme was alkaline phosphatase (n = 11). Although total bilirubin was elevated in only about 10 individuals, the direct component of bilirubin was risen above the normal range in one-thirds of the study population, and more so in severe patients (p = 0.005). Fulminant hepatic failure was recorded in two of the patients with severely deranged liver enzymes. An ROC curve for hepatic was highly predictive for AST (AUC: 0.767) and total bilirubin (AUC: 0.752) in non-surviving patients at sensitivity of 87% and PPV of 94-95%, respectively.

6) Inflammatory and Biochemical Markers

C-reactive protein, serum ferritin, and LDH levels were found elevated in almost 90% of the severe cases in our study, while around 70% in non-severe patients as well. CRP was found more predictory for ICU admission with a cut-off value at 103.60 mg/L, having a sensitivity of 81%, specificity of 73%, and PPV of 85% (AUC: 0.806, p < 0.001). While LDH was predicting mortality at a cut-off value of 574.50 U/L, with a sensitivity of 91%, a specificity of 74%, and PPV of 97% (AUC: 0.887, p < 0.001). Serum ferritin was less predictable for the severity of the disease as compared to the above entities.

Both procalcitonin and D-dimer were slightly more affected in severe cases but not statistically significant. Erythrocyte sedimentation rate (ESR) was non-specifically elevated in almost all patients. With respect to the cardiovascular comorbidities in a number of study individuals, Troponin I and Pro-BNP were also found elevated in most of those individuals; however, elevation did not show correlation with either ICU stay or mortality. Figure 4 shows the in-hospital progression of biochemical labs in the study population.

## Discussion

In our study, the age of individuals infected from novel coronavirus disease (COVID-19) ranged from 51-75 years with a mean age of 55.8 years coinciding with outcomes of various studies, indicating that this novel disease commonly infects middle-aged and older population [3-4, 8-9, 11-12, 14]. Male gender was predominantly more exposed and affected as compared to the female population evaluated in our study results synchronizing with findings of multiple studies while contrasting with the results of a few [3]. Comorbidities encountered within the elder population evaluated in our study are diabetes mellitus (DM), hypertension (HTN), ischemic heart disease (IHD), and chronic kidney disease (CKD) projecting towards poor prognosis of disease correlating with outcomes of countable studies [3, 9, 11-12]. Elder population of our sample size developed damages and failures of vital organs such as acute liver injury, renal failure, respiratory failure, septic shock, while fulminant liver failure and coagulopathy being rare manifestations coinciding with results of few studies [3-5, 12]. Frequent ARDS was found in studies from China, as opposed to our study [12, 14]. Acute cardiac injuries were found more frequently in a study as compared to renal injury, a finding contradicting our study with more renal involvement secondary to sepsis [12]. Coagulopathy and septic shock were less frequent complications in a study, similar to our findings but the later was more frequently occurred in our population [14].

Clinical manifestations encountered by sufferers evaluated in our study were fever, dry cough and shortness of breath being predominant ones; apart from this sore throat, fatigue, diarrhea, muscle and joint pains, nasal congestion/rhinorrhea, pleurisy, vomiting, headache, nausea, abdominal pain, diminished appetite are also encountered - these manifestations synchronize with manifestations reported in several studies [7, 11-12, 14]. The average length of hospital stay observed within the population of our sample size was eight days coinciding with the findings of very few studies [12, 14]. Laboratory parameters detected within sufferers in our study population were lymphocytopenia, leukopenia, thrombocytopenia, deranged liver function enzyme levels, elevated CRP, and D-dimers coinciding with results of numerous studies [11-12, 14]. Sufferers observed in the sample population of our study contacted infection primarily by respiratory droplets of other infected individuals thus local transmission, finding coinciding with outcomes of a number of studies [4, 5, 11-12]. Radiographs were frequently showing bilateral patchy or interstitial shadowing followed by ground-glass appearance, similar to another study in Wuhan, China [12]. While another study with an extensive sample size showed bilateral shadowing most frequent findings followed by ground-glass appearance, and interstitial infiltrate being less common finding as opposed to our study [14].

In our study, hemoglobin levels detected in sufferers of coronavirus disease (COVID-19) admitted in the wards and those who recovered are slightly different (Increased) as compared to patients who were admitted in intensive care units (ICU) and deceased outcome correlating with findings of limited studies [14, 17]. High counts of neutrophils and elevated total leukocyte counts (TLC) were detected within patients admitted in ICU and those deceased as compared to patients admitted in the ward and those who recovered - findings consistent with detections of multiple studies [18-21]. In contrast, we detected significantly declining levels of lymphocytes (lymphocytopenia) in critically infected patients or deceased ones as compared to patients with non-severe disease synchronizing with outcomes of various studies [18-20]. Interestingly in our study, less frequency of thrombocytopenia was detected in patients admitted in ICU while the majority was detected with a normal range of platelets as compared to significant thrombocytopenia within severe patients in another study conducted in similar pattern contrasting the outcome of our study [19]. Another study also contrasted with our outcomes based on platelet count, detecting slightly elevated levels of platelets in patients with mild disease as compared to severe patients [22]. One study depicted a slight difference in counts of platelets in both mild and severe disease as compared to significantly elevated levels of platelets in severe disease within our study [18].

In outcomes of our study, renal parameter creatinine was detected within normal ranges in patients admitted in wards with mild disease or patients who recovered and significantly elevated creatinine was detected in patients admitted in ICU and those expired - a finding consistent with multiple studies [4, 14, 17-18]. The sodium levels in patients evaluated in our sample size reported normal ranges in severe vs. non-severe, as compared to another study conducted reporting a decrease in levels of sodium in patients admitted in intensive care units thus contrasting our findings, although during the hospital stay severe patients developed markedly increased sodium levels in our study (Figure 4e) [18]. Elevated levels of potassium but within the normal range were detected in patients of ICU and deceased ones as compared to ones admitted in the ward or recovered, outcome correlating with another study quoting similar outcomes [18]. In our study elevated levels of urea and chloride, decreased serum bicarbonate levels were reported in patients of intensive care units and deceased correlating with outcomes of negligible studies [14, 23].

Elevated levels of alanine transaminase (ALT), aspartate aminotransferase (AST) and gamma-glutamyl transferase (GGT) and slightly elevated levels of total bilirubin were detected in patients of intensive care units and deceased as compared to patients of wards or those recovered - findings synchronizing with outcomes of various studies [24-26]. Elevated levels of inflammatory parameters C-reactive protein (CRP), lactate dehydrogenase (LDH), D-dimer, erythrocyte sedimentation rate (ESR), ferritin and procalcitonin (PCT) were detected in patients admitted in ICU, outcomes also coinciding with detections of multiple studies [14, 17-21]. An increased NLR value has been shown associated with worse prognosis of COVID infection in multiple studies, a finding similar to our study [17-21, 23]. A study showed PLR value of 180 as a potential marker for severity of disease with AUC of 0.784, having 77% sensitivity and 44% specificity [19]. This finding was slightly underwhelmed in our study with a lower AUC at a cut-off value of 153.65 having 72% sensitivity and 65% specificity. Another study depicted PLR as a severity marker only when considered at peak platelet levels during hospital stay [22]. A low LMR and LCR also correlated with disease severity in our study, a finding which is seconded by two separate studies [27-28].

There were a few limitations in our study, the one being a relatively small sample size than previous Chinese studies, a retrospective analysis means all laboratory parameters were not continuously monitored in every patient. Another limitation is, the radiological investigation used only was a chest radiograph and no CT scan was performed to confirm the radiological finding, as has been postulated more diagnostic test for Covid-19. Lastly, follow-up labs were not readily available for every patient, especially for patients sent for home isolation.

## Conclusions

A single centered study of 100 hospitalized patients in a developing country with confirmed COVID-19 showed a number of factors linked with disease severity and mortality along with dynamic changes of the laboratory investigations during hospital stay affecting prognosis. The clinical profile and characteristics of patients along with contrasting levels of several biomarkers can be a predictor of disease severity and mortality, although multiple large center prospective surveys must undergo in order to explore the findings demonstrated. In our study, we found several markers including elevated leukocyte and neutrophil counts, NLR, CRP, LDH, and deranged urea/creatinine most likely associated factors linked with disease severity and mortality. While lymphopenia, decreased LMR, LCR, and bicarbonate levels also correlate with disease severity. ARDS was a relatively lesser-known finding in our population, on the other hand, acute liver and kidney injuries are more frequent in the sufferers of COVID-19.
