# Correction: Clinical Profiles, Characteristics, and Outcomes of the First 100 Admitted COVID-19 Patients in Pakistan: A Single-Center Retrospective Study in a Tertiary Care Hospital of Karachi

**DOI:** 10.7759/cureus.c34

**Published:** 2020-08-06

**Authors:** Muhammad Sohaib Asghar, Syed Jawad Haider Kazmi, Noman Ahmed Khan, Mohammed Akram, Salman Ahmed Khan, Uzma Rasheed, Maira Hassan, Gul Muhammad Memon

**Affiliations:** 1 Internal Medicine, Dow International Medical College, Dow University Hospital, Dow University of Health Sciences, Karachi, PAK; 2 Emergency Medicine, Liaquat National Hospital and Medical College, Karachi, PAK; 3 General Surgery, Liaquat National Hospital and Medical College, Karachi, PAK; 4 Internal Medicine, Liaquat National Hospital and Medical College, Karachi, PAK; 5 Internal Medicine, Dow International Medical College, Karachi, PAK

Tables 3 and 5 included the following incorrect data. These data points have been corrected.

- Table 3: In the serum urea section, the mean in ICU stay is 58.33 ± 37.22. The standard deviation was originally incorrectly entered as 1.94 by the authors and has been replaced with 37.22.
- Table 5: In the basophil section, the cut off in death is 0.50. The value was originally incorrectly entered as 655.00 by the authors and has been replaced with 0.50.

